# Exploring mean kurtosis in MR diffusion kurtosis imaging for early detection of lumbar spine degeneration: a systematic review

**DOI:** 10.12688/f1000research.163638.2

**Published:** 2025-09-01

**Authors:** Neil Abraham Barnes, Winniecia Dkhar, Rajagopal Kadavigere, Suresh Sukumar, Abhimanyu Pradhan, Priya P.S., Vaishali K

**Affiliations:** 1 Department of Medical Imaging Technology, Manipal College of Health Professions, Manipal Academy of Higher Education, Manipal, Karnataka, 576104, India; 2 Department of Radiodiagnosis and Imaging, Kasturba Medical College, Manipal Academy of Higher Education, Manipal, Karnataka, 576104, India; 3 Department of Physiotherapy, Manipal College of Health Professions, Manipal Academy of Higher Education, Manipal, Karnataka, 576104, India

**Keywords:** Magnetic Resonance Imaging, Diffusion Kurtosis Imaging, Intervertebral disc space, Degenerative Spine

## Abstract

**Objectives:**

Degenerative lumbar spine disease, a leading cause of chronic pain and disability in older adults, results from the progressive degeneration of intervertebral discs. This systematic review evaluates the role of mean kurtosis (MK) as a diffusion kurtosis imaging (DKI) parameter in the early diagnosis of degenerative spine disease and its potential to enhance patient outcomes.

**Methods:**

A systematic review was conducted following PRISMA guidelines, with a comprehensive search yielding 7,290 articles. After screening, three studies met the inclusion criteria. Quality assessment was performed using the QUADAS tool, considering studies with a score of ≥10 as high-quality. Data extraction focused on DKI parameters, particularly MK, in assessing early disc degeneration. The study was registered in PROSPERO (CRD42024554902) on June 5, 2024.

**Results:**

Findings indicate that MK plays a crucial role in detecting microstructural changes in the intervertebral disc space of the lumbar spine. These changes closely correlate with clinical symptoms and the extent of degeneration observed on conventional MRI. DKI-derived MK appears to offer greater sensitivity in identifying early-stage microstructural degeneration compared to traditional imaging methods

**Conclusions:**

MR DKI demonstrates significant potential for detecting subtle, early changes in lumbar spine degeneration. Integrating DKI into clinical practice could enhance diagnostic accuracy, enable earlier interventions, and ultimately improve patient outcomes.

## Introduction

Degenerative diseases are widespread, affecting a significant portion of the population. Lumbar spine degeneration is a prevalent condition that significantly impacts adults, causing chronic low back pain (CLBP) and reduced mobility. ^1^ The World Health Organization (WHO) has classified CLBP as one of the major causes of disability globally, contributing substantially to both health care and economic burdens. ^2^ Degeneration changes in the intervertebral disc space impact the structural integrity, leading to the bulging of the nucleus pulposus (NP) and the annulus fibrosus (AF) tearing. These changes lead to spinal stenosis and reduce the stability of the spine. ^3^ Consequently, this results in decreased intervertebral disc height and spinal abnormalities, impairing spinal functionality and restricting motion. Degenerative changes in the spine are diagnosed at more advanced stages, when they become clinically apparent, limiting the effectiveness of the treatment and negatively patient outcomes. Early diagnosis is essential, as it allows for more effective interventions and facilitates the development of personalized, targeted therapies, thereby improving clinical outcomes and enhancing the overall quality of life. ^4, 5^

Magnetic resonance imaging (MRI) is currently the gold standard in diagnosing degenerative spine diseases, where T1 and T2 weighted sequences are commonly used in detecting structural abnormalities. ^6^ Conventional imaging sequences are limited to macroscopic diagnostic changes, and therefore unable to detect subtle microstructural changes. This limitation has driven the development of more advanced MRI applications such as Diffusion Weighted Imaging (DWI) and its application such as Diffusion tensor Imaging (DTI) and DKI. ^7, 8^

DKI estimates kurtosis-tensor-specific parameters including MK, axial kurtosis (AK), radial kurtosis (RK), mean diffusivity (MD), axial diffusivity (AD), and radial diffusivity (RD). DKI parameter maps, such as MK, are directly derived from the dMRI data and often have implausible values without any additional processing. Empirical findings from biological tissues and fundamental physical principles indicate that MK should maintain a positive value and remain below 3. ^9– 11^ Many visually implausible voxels exhibit MK values that fall outside the typical range of 0–3. However, many of these implausible voxels remain within the empirically defined range, yet still appear unusually dark or bright compared to their surrounding. ^12, 13^ MK is derived from DKI, an advanced MRI technique that extends Diffusion Tensor Imaging (DTI) by accounting for non-Gaussian water diffusion in biological tissues. Unlike DTI, which assumes unrestricted diffusion, MK captures microstructural complexity by measuring the degree of diffusion heterogeneity caused by collagen fibers, cellular membranes, and extracellular matrix components. In the intervertebral disc, degeneration leads to proteoglycan loss, increased collagen cross-linking, and fiber disorganization, causing higher MK values. Similarly, in the annulus fibrosus (AF), disruptions in fiber integrity alter diffusion patterns. These properties make MK a sensitive biomarker for early degenerative changes, detecting microstructural alterations before they become apparent on conventional MRI. ^14– 16^

In addition to its applications in spinal imaging, DKI has demonstrated broad diagnostic utility across various fields, including neuroimaging, oncology (liver, breast, renal, and prostate cancers), musculoskeletal, and abdominal imaging. Its ability to detect early microstructural changes has made it a crucial tool for accurate diagnosis, prognostication, and treatment planning. ^17– 22^

DKI and its parameter MK are key techniques for detecting early microstructural changes in the spine. This review aims to evaluate MK’s role in detecting the early diagnosis of degenerative spine disease and assess its effectiveness in improving patient outcomes.

## Methods

### Literature search

An extensive search was conducted across the Scopus, Embase, Web of Science, and PubMed databases for studies published between January 2010 and 2024. To identify additional relevant research papers, a thorough manual cross-checking of the reference lists from all retrieved articles was performed. The database searches utilized the following clinical concern headings and medical keywords: “Diffusion Kurtosis Imaging,” “DKI,” “Magnetic Resonance Imaging,” “MRI,” “Degenerative Spinal Disease,” and “Degenerative Disease.” The study has been registered in PROSPERO under the registration number CRD42024554902 on June 5, 2024.

### Selection criteria

The inclusion criteria for the systematic review were as follows: (a) studies utilizing a 3T MRI scanner, (b) research conducted exclusively on human subjects, (c) MRI sequences specifically involving DKI, (d) studies that report DKI parameters, (e) a focus on degenerative lumbar spine conditions, (f ) publications with verified findings in peer-reviewed journals, and (g) studies published in English. Articles were selected based on the guidelines for systematic reviews of diagnostic tests. ^23^ With an initial screening of titles and abstracts to ensure adherence to both inclusion and exclusion criteria. The subsequent step involved a comprehensive assessment of the full texts of the articles, applying the same inclusion and exclusion criteria to finalize the studies to be included in the systematic review. Excluded articles were those that did not report DKI parameters, focused on neurodegenerative diseases, oncology, musculoskeletal diseases, cervical spine studies, studies done on CT and X-ray, and studies that did not have any MRI data, studies unrelated to diagnostic performance, or were preclinical studies. Additionally, case reports, letters, review articles, unpublished articles, and comments were excluded from consideration.

### Data extraction

In this systematic review, we extracted descriptive data from the included studies to assess the efficacy of Mean Kurtosis (MK) in detecting early degenerative changes in the nucleus pulposus (NP) and annulus fibrosus (AF) of the lumbar intervertebral disc. All included studies, published between 2010 and 2024, were conducted by researchers in China and Japan and predominantly followed prospective cohort designs. MRI scanners from major manufacturers, such as the GE Discovery MR750 (3T) and Philips Achieva (3T), were used, with technical parameters like repetition time (TR) ranging from 2000 to 10,758 ms and echo time (TE) between 71.7 and 96 ms. Diffusion gradients (b-values) varied across the studies, with common values of 0, 1000, and 2000 s/mm ^2^, and the number of diffusion directions ranged from 6 to 30. The mean age of participants across the studies was approximately 44 years, with 160 subjects. Mean MK values for healthy lumbar discs ranged from 0.65 to 1.99, while degenerated discs showed elevated MK values between 1.4 and 1.7, indicating significant microstructural changes. These findings highlight the potential of MK as a reliable biomarker for early disc degeneration, with reported sensitivities and specificities around 70-90% in detecting degeneration, underscoring the clinical utility of MK in spinal pathology evaluation. In this study the data extraction was performed by two reviewers (NAB) and (WD) and the third reviewer (RK) evaluated it independently. The extracted data are presented in Tables 1 and 2 *.*

**Table 1.** Overview of the studies included in the systematic review, detailing study design, sample size, imaging protocol, and key findings related to MK values in lumbar spine degeneration.

Author name, Country, Year	Study design	MR scanner	Mean Age	No. of subjects	Technical parameters	b values	No. of directions
Feifei Zeng et al. (China, 2019)	P	GE Discovery MR750, 3T	42.17	75	TR- 2000 ms TE- 71.7 ms	0, 1000, and 2000 s/mm ^2^	30
Li Li et al. (China, 2019)	P	GE Discovery MR 750, 3T	32.13	53	TR- 2500 ms TE- 96 ms	0, 1250, and 2500 mm/s ^2^	25
Takano et al. (Japan, 2020)	P	Philips, Achieva, 3T	57.7	32	TR- 10,758 ms TE- 88 ms	0, 700, 1400, and 2100 s/mm ^2^	6

TR- time of Recovery, TE- Time of Echo.

**Table 2.** Comparison of Mean Kurtosis Values Across Studies A comparative analysis of MK values reported in different studies, highlighting variations in degenerative stages and correlation with clinical symptom.

Author	Mean ± SD MK NP (Healthy)	Mean ± SD MK NP (Degeneration)	Mean ± SD MK AF (Healthy)	Mean ± SD MK AF (Degeneration)	No. of Discs	Sensitivity	Specificity
Feifei Zeng et al.	0.67 ± 0.04	1.44 ± 0.19	1.285 ± 0.20	1.91 ± 0.32	369	79.42%	79.3%
Li Li et al.	0.65 ± 0.13	0.63 ± 0.12	NR	NR	53	90%	70%
Takano et al.	1.994	1.743	NR	NR	96	NR	NR

MK- Mean Kurtosis, NP-Nucleus Pulposus, AF- Annulus Fibrosus, NR- No Results.

### Quality assessment

All studies were meticulously reviewed. Study selection and data extraction were conducted independently by two reviewers. Quality assessment and eligibility were evaluated using the QUADAS tool, ^24^ a validated instrument specifically designed for systematic reviews of diagnostic accuracy studies. Three broad domains were considered: (1) Bias, scored from 0 to 9; (2) Applicability, scored from 0 to 2; and (3) Reporting quality, scored from 0 to 3. The total QUADAS score ranged from 0 to 16, with a score of ≥10 indicating high methodological quality. To resolve discrepancies between the two reviewers, a third reviewer re-evaluated all items, and consensus was reached through discussion. The QUADAS tool, comprising 14 quality items, was used to assess each study. Each item was rated as 'Yes,' 'No,' or 'Unclear,' with a maximum possible score of 14.

## Results

### Study selection and quality assessment

**Figure 1.**
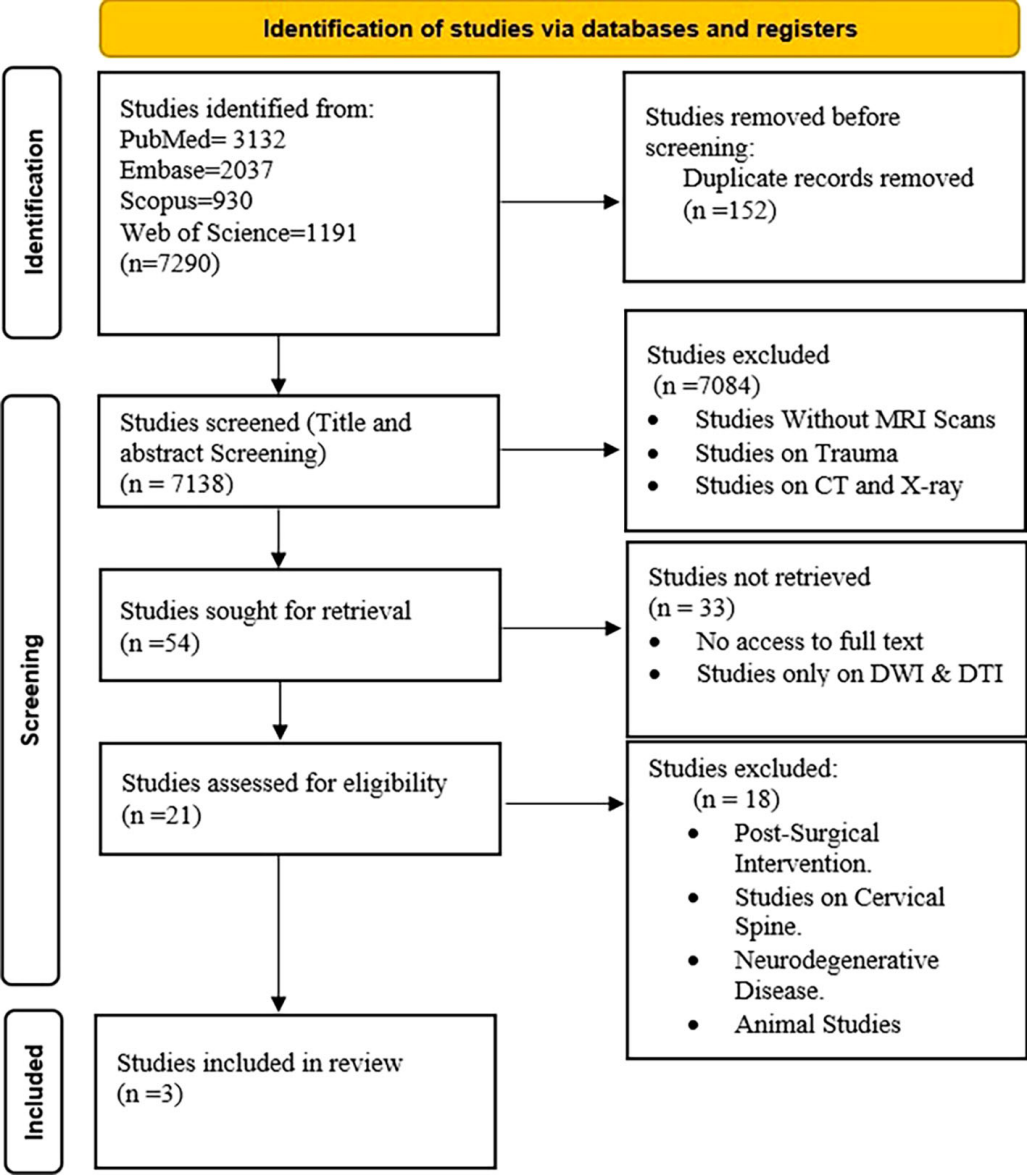
PRISMA Flowchart of Study Selection A flowchart depicting the systematic review process for evaluating the role of MK in lumbar spine degeneration. The initial database search identified 7290 studies, of which 3 were included in the final review following eligibility criteria.

The systematic search initially identified 7290 potential studies, of which only 3 met the inclusion criteria for this review ( Figure 1). All included studies were published between 2010 and 2024. The QUADAS tool quality assessment revealed scores ranging from 11 to 14. Items 9, 10, and 11 of the QUADAS tool, about the clarity of the reference standard execution, the translation effects of the reference standard without index test results, and the translation of index test results without reference standard information, respectively, were identified as areas of uncertainty in many of the included studies.

### Study characteristics

The three included studies involved a total of 160 patients who underwent MRI with a DKI sequence, resulting in the diagnosis of 518 discs and the calculation of DKI parameters. All studies employed a prospective study design and were conducted using 3 Tesla MRI scanners. Detailed information regarding the subjects with degenerative disc disease of the lumbar spine is characterized and summarized in Tables 1 and 2.

### Characteristics of MK in evaluating the NP

All three studies reported MK values for the NP, showing variability across healthy and degenerated discs. Zeng et al. ^25^ found an increase in MK from 0.67 in healthy NP to 1.44 in degenerated NP. In contrast, Li et al. ^26^ observed minimal change, with MK values of 0.65 in healthy NP and 0.63 in degenerated NP. Takano et al. ^27^ reported higher baseline MK values, with 1.994 in healthy NP, slightly decreasing to 1.743 in degeneration. These variations reflect differences in study design and patient characteristics. The mean values of MK in NP in the study by Zeng et al. ^25^ were reported based on correlating the by taking Pfirrmann grading scale 1 for healthy disc and grade 4 for degenerative disc.

### Characteristics of MK in evaluating the AF

Zeng et al. ^25^ provided MK values for the AF. In healthy AF, the mean MK was 1.285, rising to 1.91 in degenerated discs, suggesting significant microstructural changes in this region during early degeneration. The MK value of AF was taken from AAF and PAF average taken and Pfirrmann grading scale 1 for healthy disc and grade 4 for degenerative disc.

### Sensitivity and specificity

Zeng et al. ^25^ demonstrated a sensitivity of 79.42% and specificity of 79.3%, indicating moderate diagnostic performance in distinguishing between healthy and degenerated discs. Li et al. ^26^ reported a higher sensitivity of 90% but a lower specificity of 70%, suggesting that while MK is a sensitive marker for early degeneration, its specificity might be lower in differentiating early-stage changes from other conditions.

## Discussion

The systematic review was conducted following PRISMA guidelines in which a total of 7260 articles were screened and three studies were included in the review based on their relevance.

The findings of the review emphasize the role of MK as a biomarker for detecting early degenerative changes in the lumbar intervertebral disc. MK, derived from DKI, extends beyond DTI by focusing on the non-Gaussian diffusion properties of water molecules, offering a deeper understanding of tissue complexity and microstructural integrity. Lumbar degeneration is marked by progressive loss of disc hydration, a reduction in proteoglycan content, and collagen fiber degradation. MK sensitively captures these early-stage macrostructural changes, quantifying the degree of water diffusion restriction, which is influenced by the heterogeneity and complexity of tissue microstructure. In a healthy intervertebral disc, the NP is highly hydrated, allowing for unrestricted diffusion. However, in degenerative discs, the loss of proteoglycans and increased collagen fiber cross-linking lead to higher diffusion heterogeneity, which in turn increases MK values. ^28– 30^

The systematic review findings suggested the variability of the MK in the NP and AF in the healthy and degenerative lumbar spine. Zeng et al., ^25^ reported a significant increase in the MK for both NP and AF in the degenerative spine. They suggested that the MK could be a useful biomarker for the early microstructural changes in the spine. Similarly, Li et al., ^26^ stated that there were limited changes in MK values of the Healthy population selected in their study. Despite this, the degenerative population in the NP region of the discs showed minimal change. In contrast, Takano et al., ^27^ reflected in their research on higher baseline MK values, with lesser in degenerative disease. These inconsistencies likely reflect the differences in the parameters, study populations, and methodological approach, highlighting the need to standardize the imaging protocol to enhance the compatibility of the findings across the studies.

The review further analyzed the sensitivity and specificity which indicate that the diagnostic performance of the DKI varied across the studies where Zeng et al., ^25^ demonstrated moderate sensitivity and specificity. In contrast, Li et al., ^26^ demonstrated higher sensitivity and lower specificity, suggesting that DKI may be highly sensitive to early degenerative changes but may face challenges in specificity.

MK demonstrated it ability to detect the early microstructural changes in the spine, while T2- T2-weighted MRI remains the gold standard for assessing disc hydration but it cannot detect the microstructural changes. ^31^ Similarly, T2 mapping and relaxometry techniques provide the ability to detect proteoglycan loss but it is limited to assess the complexity of the fiber tract integrity in the AF. ^32, 33^

Although this review primarily focused on lumbar degeneration, the applicability of MK extends to other spinal regions. For instance, studies on cervical spondylotic myelopathy (CSM) have shown significantly lower MK values in stenotic segments, suggesting that MK is not only sensitive to disc degeneration but also to gray and white matter disruptions caused by chronic compression. Notably, MK values in the gray matter correlate with Japanese Orthopaedic Association (JOA) scores, reinforcing its potential role as a clinical indicator of neurological impairment. ^34– 36^

The clinical implications of MK in spinal pathology are significant. First, MK can facilitate early diagnosis, potentially allowing for preventive interventions before severe structural damage occurs. Second, MK may serve as a biomarker for monitoring disease progression and guiding treatment decisions in conservative vs. surgical management. Lastly, its role in detecting microstructural damage in neural tissues suggests that MK may be useful in assessing post-surgical recovery or evaluating regenerative therapies such as platelet-rich plasma (PRP) or stem cell injections. This review emphasizes the importance of early detection and management of degenerative spine in line with Sustainable Development Goal 3 (good health and well-being). There is concern about this condition globally, especially among aging populations, which gradually reduces quality of life and increases health care costs. DKI plays a vital role in early diagnosis, of the degenerative spine and hence, thereby reducing healthcare costs, improving patient outcomes, promoting global health equity, and improving individual quality of life.

### Limitations and future recommendations

While MK has demonstrated potential as a biomarker for detecting early degenerative changes in the spine, several limitations must be addressed before it can be widely adopted in clinical practice. A key challenge is the lack of standardized imaging protocols, including variations in b-value selection, post-processing methods, and segmentation techniques, which contribute to inconsistencies in reported MK values and hinder cross-study comparability. Additionally, small sample sizes and heterogeneous study populations limit the generalizability of findings, necessitating larger, multi-center studies with diverse patient cohorts. Another critical gap is the absence of longitudinal data, making it unclear whether MK can reliably track disease progression or predict treatment outcomes. Although MK offers insights into microstructural complexity, its comparative efficacy against established imaging modalities, such as T2 mapping, DTI, and quantitative susceptibility mapping (QSM), remains underexplored. Furthermore, current MK quantification relies on manual or semi-automated segmentation methods, introducing inter-observer variability; integrating AI-driven image analysis could enhance reproducibility and clinical feasibility. To overcome these challenges, future research should prioritize standardizing diffusion imaging protocols, conducting longitudinal studies to evaluate MK as a predictive biomarker, and exploring multi-parametric MRI approaches that combine MK with T2 relaxometry, ultrashort echo-time (UTE) MRI, and QSM for a more comprehensive assessment of spinal degeneration. Additionally, the integration of machine learning algorithms for automated MK quantification could improve diagnostic accuracy and streamline clinical implementation. Addressing these limitations will be essential in establishing MK as a robust and clinically viable tool for assessing spinal pathology across multiple regions.

## Conclusion

The systematic review helps in identifying the diagnostic importance of the advanced MRI applications such as DKI and its parameters MK which played a vital role in the early diagnosis of the degenerative spine and subtle microstructural change that occurs in the intervertebral disc space of the lumbar spine. The review focused on the effectiveness of the MK in the detection of early degenerative in the degenerative lumbar spine. Integrating MK into routine diagnostic workflows could significantly enhance the accuracy of detecting lumbar spine degeneration, enabling timely interventions. This advancement has the potential to reduce healthcare costs, improve patient outcomes, and contribute to global health objectives aimed at promoting better quality of life through early and effective management of degenerative spine conditions.

## Ethics and consent

Ethical approval and consent were not required.

## Data Availability

No data are associated with this article. Figshare: The PRISMA checklist and the flowChart for “Exploring mean kurtosis in MR diffusion kurtosis imaging for early detection of lumbar spine degeneration: a systematic review”. DOI: **https://doi.org/10.6084/m9.figshare.28695512.v2** ^37^ Data are available under the terms of the Creative Commons Attribution 4.0 International license (CC-BY 4.0).
