# Supplementary material for: Exploring mean kurtosis in MR diffusion kurtosis imaging for early detection of lumbar spine degeneration: a systematic review
Source: F1000Res. 2025 Sep 1;14:440. Originally published 2025 Apr 15. [Version 2] doi: 10.12688/f1000research.163638.2 (PMC12374150; doi:10.12688/f1000research.163638.2)
Supplement: Peer review history

# Reviewer report: Reviewer response for version 1

**DOI:** 10.5256/f1000research.180025.r398769

**Published:** 2025-08-22

**Authors:** Manna Debnath

**Affiliations:** 1 Pramukhswami Medical College and Shree Krishna Hospital, Bhaikaka University, Anand, Gujarat, India

Reviewers comments:

 1. The abstract is well written, highlighting this research article's overall summary.

 2. In the Methods section, you stated that the literature search covered studies published between January 2010 and 2024, and in data extraction, you included studies from 2019 and 2020. However, in the Results section, the second line mentions that all included studies were published between 2010 and 2024. Please clarify this for better understanding by the reader.

 3. In the Results section, Figure 1 shows all the databases from which you derived the articles (n = 7290), and then removed duplicate records during screening (n = 152). If you remove 152 studies from 7290, the total should be 7138. However, in the next step, you mention that 7140 studies were screened, including title and abstract. Please verify the PRISMA flowchart.

 4. The overall article is technically sound and scientifically robust, and it will definitely contribute to spinal degeneration imaging.

Are the rationale for, and objectives of, the Systematic Review clearly stated?

Yes

Is the statistical analysis and its interpretation appropriate?

Yes

If this is a Living Systematic Review, is the ‘living’ method appropriate and is the search schedule clearly defined and justified? (‘Living Systematic Review’ or a variation of this term should be included in the title.)

Yes

Are sufficient details of the methods and analysis provided to allow replication by others?

Yes

Are the conclusions drawn adequately supported by the results presented in the review?

Yes

Reviewer Expertise:

Magnetic Resonance Imaging (MRI)

I confirm that I have read this submission and believe that I have an appropriate level of expertise to confirm that it is of an acceptable scientific standard.

# Reviewer report: Reviewer response for version 1

**DOI:** 10.5256/f1000research.180025.r384270

**Published:** 2025-05-28

**Authors:** Priyanka Chandrasekhar

**Affiliations:** 1 Apollo University, Chittoor, India

**Summary of the Article:**

 This systematic review evaluates the effectiveness of mean kurtosis (MK) derived from diffusion kurtosis imaging (DKI) as a biomarker for early detection of intervertebral disc degeneration (IDD) in the lumbar spine. The authors screened 112 studies and included three high-quality studies based on specific inclusion criteria. The review concludes that MK shows promise in detecting early microstructural changes in the intervertebral discs before morphological degeneration becomes apparent in conventional imaging, offering significant clinical potential for early diagnosis and intervention.

**Final Comments:**

 This is a well-structured and clearly written systematic review that presents a novel application of diffusion kurtosis imaging in spinal diagnostics. The methodology is sound, the synthesis of data is logical, and the conclusions are supported by the findings. The article is scientifically robust and contributes meaningfully to the field of spinal imaging.

Are the rationale for, and objectives of, the Systematic Review clearly stated?

Yes

Is the statistical analysis and its interpretation appropriate?

Not applicable

If this is a Living Systematic Review, is the ‘living’ method appropriate and is the search schedule clearly defined and justified? (‘Living Systematic Review’ or a variation of this term should be included in the title.)

Not applicable

Are sufficient details of the methods and analysis provided to allow replication by others?

Yes

Are the conclusions drawn adequately supported by the results presented in the review?

Yes

Reviewer Expertise:

Medical Imaging Technology, Foetal MRI, Computer Tomography, Magnetic Resonance Imaging.

I confirm that I have read this submission and believe that I have an appropriate level of expertise to confirm that it is of an acceptable scientific standard.
